# Long-term follow-up of full-arch immediate implant-supported restorations in edentulous jaws: a clinical study

**DOI:** 10.1186/s40729-020-00232-8

**Published:** 2020-07-30

**Authors:** Laura Werbelow, Michael Weiss, Alexander Schramm

**Affiliations:** 1 Department of Oral and Maxillofacial Surgery, University Medical Center Hamburg-Eppendorf, Martinistraße 52, 20246 Hamburg, Germany; 2 OPUS Dental Clinic, Neue Straße 72-74, 89073 Ulm, Germany; 3 Department of Oral and Plastic Maxillofacial Surgery, Military Hospital Ulm, Oberer Eselsberg 40, 89081 Ulm, Germany; 4 Department of Oral and Maxillofacial Surgery, University Hospital Ulm, Albert-Einstein-Allee 11, 89081 Ulm, Germany

**Keywords:** Full-arch immediate-loading implant dental rehabilitation

## Abstract

**Background:**

This study aims to show the long-time stability of straight and tilted implants loaded immediately with a provisional resin bridge followed by a definitive prosthodontic rehabilitation in edentulous jaws despite difficult hygiene conditions postoperatively.

**Results:**

This study included the participation of 23 patients and the restoration of 170 dental implants in 32 edentulous jaws. Patient data was analyzed from the start of treatment with a minimum follow-up period of 6 years in order to determine long-term implant success rates. However, the age of patients at time of surgery significantly affected the BOP to the detriment of younger patients (median 62 years old).

**Conclusion:**

Although there was a higher risk of implant failure due to general disease, all the implants in this study survived successfully. As a replacement for a complete dental arch, the reduced number of implants in combination with the avoidance of augmentations reduces treatment costs. The immediate fixed prosthetic restoration of edentulous jaws thus represents a reliable therapeutic alternative to a two- to three-stage procedure. Optimized aftercare including professional teeth cleaning (PTC) (at least twice a year) can minimize the anamnestic effect of smoking, diabetes mellitus, and osteoporosis on BOP and possible bone loss.

## Introduction

Being threatened by complete edentulism has a negative impact on patients’ quality of life. Offering an implant-supported dental rehabilitation can improve the patients’ phonetic and masticatory function and therefore quality of life [1]. The number of implants for fixed prosthodontic restoration of edentulous jaws is a discussion point among experts. Modern concepts allow a reduced number of implants, nevertheless patients normally undergo 2–3 surgical treatments until the permanent prosthetic restoration is finalized. Possible therapies are limited because of bone atrophy, rejection of big bone grafts, high treatment costs, and long treatment duration. The combination of upright implants with tilted implants can compensate the lack of phonetic and masticatory performance while avoiding bone grafting procedures and high financial costs. About 20 years ago, Paolo Malo started with this treatment concept. Since Paolo Malo started developing this treatment approach in 1993, many varieties of dental implant systems have been introduced and developed. One of these treatment concepts is the “fast&fixed”-protocol (bredentGmbH & Co. KG, Senden, Germany). This concept combines the avoidance of augmentations immediate loading of a temporary full arch resin bridge. After a 3-month healing period, patients receive their permanent prosthodontic restoration (either fixed with occlusal screws or removable prostheses attached to bars). Due to a large variety of treatment approaches, very few clinical studies have examined the long-term survival rate of immediately loaded dental implants in edentulous jaws, which are later replaced by definitive prostheses, and their correlation to anamnestic risk factors [2–4]. Therefore, the aim of this study was to evaluate the clinical outcome of implant success within the full-arch immediate loading technique in a single-center retrospective cohort study by analyzing the 6 year survival rate of the dental implant and its correlation to oral hygiene and other risk factors.

## Methods and materials

### Case selection

This single-center retrospective cohort study was conducted from April to October 2017. A total of 39 patients (20 female, 19 male) between 49 and 83 years underwent treatment in the dental clinic OPUS DC in Ulm (Germany) from 2008 to 2011. Forty-nine lower and/or upper jaws of the 39 patients were included in the study. However, 13 patients refused to participate in the study or were lost to follow-up. Three patients died before the evaluation. This study therefore included a total of 170 dental implants in 32 restored edentulous jaws in 23 patients. The patients were followed up from the start of treatment with a minimum period of 6 years in order to detect long-term implant success rates. Only patients receiving four to six dental blueSKY© implants (bredent GmbH & Co. KG, Senden, Germany) in the upper and/or lower jaw with immediate loading by a resin bridge were included in the study. Exclusion criteria included the absence of immediately fixed loading due to a lack of primary implant stability (< 30 N*cm bone insertion torque) and therefore immediately removable loading with a temporary denture after surgery and secondary fixed prosthodontic restoration. Restorations in partially edentulous jaws were also excluded from the study. Final restoration was performed after a 3 to 6 months healing period. Regularly scheduled PTC sessions happened at least twice a year postoperatively. These sessions included a basic periodontal examination, a yearly radiographic examination with a panoramic x-ray, and a professional teeth cleaning. All participating patients also completed a yearly health questionnaire. Special attention was paid to possible risk factors such as nicotine abuse, osteoporosis, and diabetes mellitus.

### Surgical protocol

All patients underwent the same surgical procedure following the “fast&fixed” protocol (bredent GmbH & Co. KG, Senden, Germany). Extraction of teeth and smoothing of bony surfaces was performed, if required, before implanting four to six dental implants. Implants in the premolar region were inserted with distal angulation to avoid sinus floor augmentations. In the premolar region of the lower jaw, implants were inserted with distal angulation to avoid damage to the mental nerve. Only implants with sufficient primary stability can be used for this protocol. Therefore, the stability of the implant was tested by the surgeon during and after insertion. The muco-periosteal flap was closed around the positioned impression copings.

### Prosthodontic protocol

After the surgical procedure, a polyether impression (Impregum©, 3 M Deutschland GmbH, Neuss, Germany) was taken, and a plaster model for the dental technician was produced. The clinician chose the definitive abutments, which compensated for the tilted implant axis, and the dental technician then created a full arch resin bridge, which was screwed in directly after manufacturing (time range from 120 min for one to 150 min for both jaws). This temporary full-arch bridge provided sufficient fixed prosthodontic restoration during a healing period of at least 3 months. Postoperative follow-up visits were scheduled closely at day 2, 7, 14, and then at least monthly until definitive restoration.

### Examination protocol

Besides the clinical examination, risk factors such as nicotine abuse, osteoporosis, diabetes mellitus, and periodontitis prior to treatment, were determined. Individual patient risk factors were recorded in their records. Detection of caries in the opposite jaw, damage or loosening of the prosthetic restoration, sensitivity probe, percussion probe, and mucosal irritations were also recorded. To avoid damaging of the peri-implant tissue and to record reliable values, a 4-point periodontal chart around every implant was recorded in millimeters with a calibrated probe (Click-Probe©, calibrated to 0, 2–0, 25 N, KerrHawe SA, Bioggio, Switzerland). The bone level around the implants was determined by evaluating postoperative and follow-up panoramic x-rays. Additionally, the angulation of the implants was also measured. Five intraoral photographs of every jaw were taken (frontal, occlusal maxilla and mandibula, lateral left, and lateral right). Plaster models of the upper and lower jaw were created, to record the present situation, e.g., prosthetic failures.

### Data extraction and outcome measurements

All data was retrieved from medical records and from the follow-up clinical examination. The data was anonymised in accordance with the World Medical Association Declaration of Helsinki (64th WMA General Assembly, October 2013).The approval of the Ethics Committee of the Landesärztekammer Baden-Württemberg was obtained (AZ F-2017-014-z). The clinical success of the implants was assessed, after a minimum period of 6 years, by comparing the radiographic bone level next to the implant and periodontal examinations of bleeding on probing.

### Statistical analysis

Parameters like gender, nicotine abuse, diabetes mellitus, osteoporosis, periodontitis preoperatively, bleeding on probing, angulation of implants, and keratinized gingiva were nominally scaled. All were described as absolute or percentage values. Two parameters were opposed in contingency tables to enable the chi-squared test for correlation. Fisher’s exact test was applied in cases of low-expected frequency. Quantitative parameters such as age, number of remaining teeth before treatment, and probing depths were described as means with standard deviation, minimum and maximum as well as quartiles. The Shapiro Wilk Test was applied to test for the normal distribution. The *U* test was applied to assess the correlation between two independent samples which were not normally distributed. Two-sided *P* values < 0.05 were considered statistically significant. No adjustments for multiple testing were performed; the results are more descriptive and explorative. All analysis was conducted using IBM SPSS Statistics 25 (SPSS Inc. an IBM Company, Chicago, IL).

## Results

### Descriptive data

**Fig. 1:**
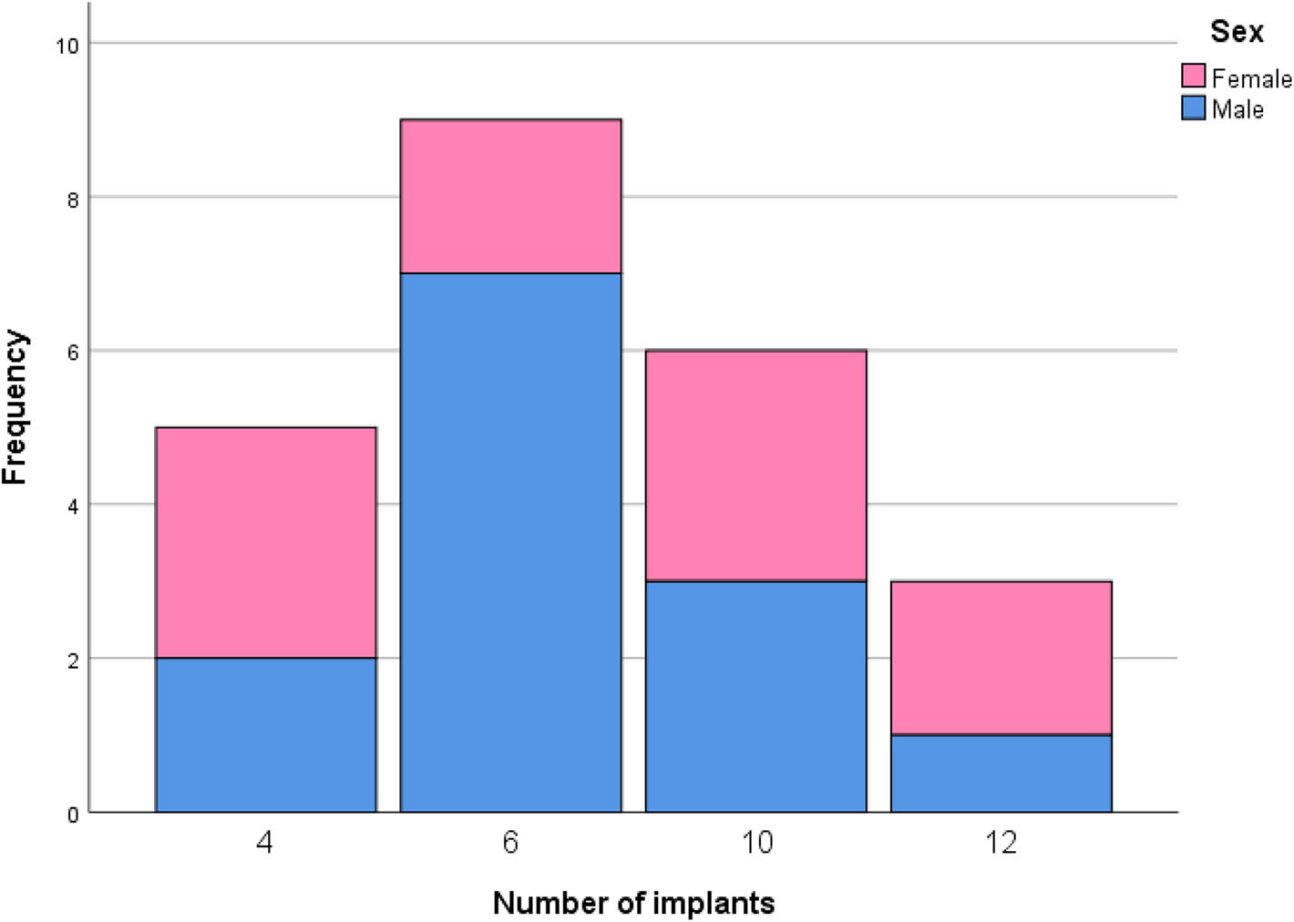
Distribution of frequency by number of implants, divided in female and male. Most patients received 6 implants (9 patients)

**Fig. 2:**
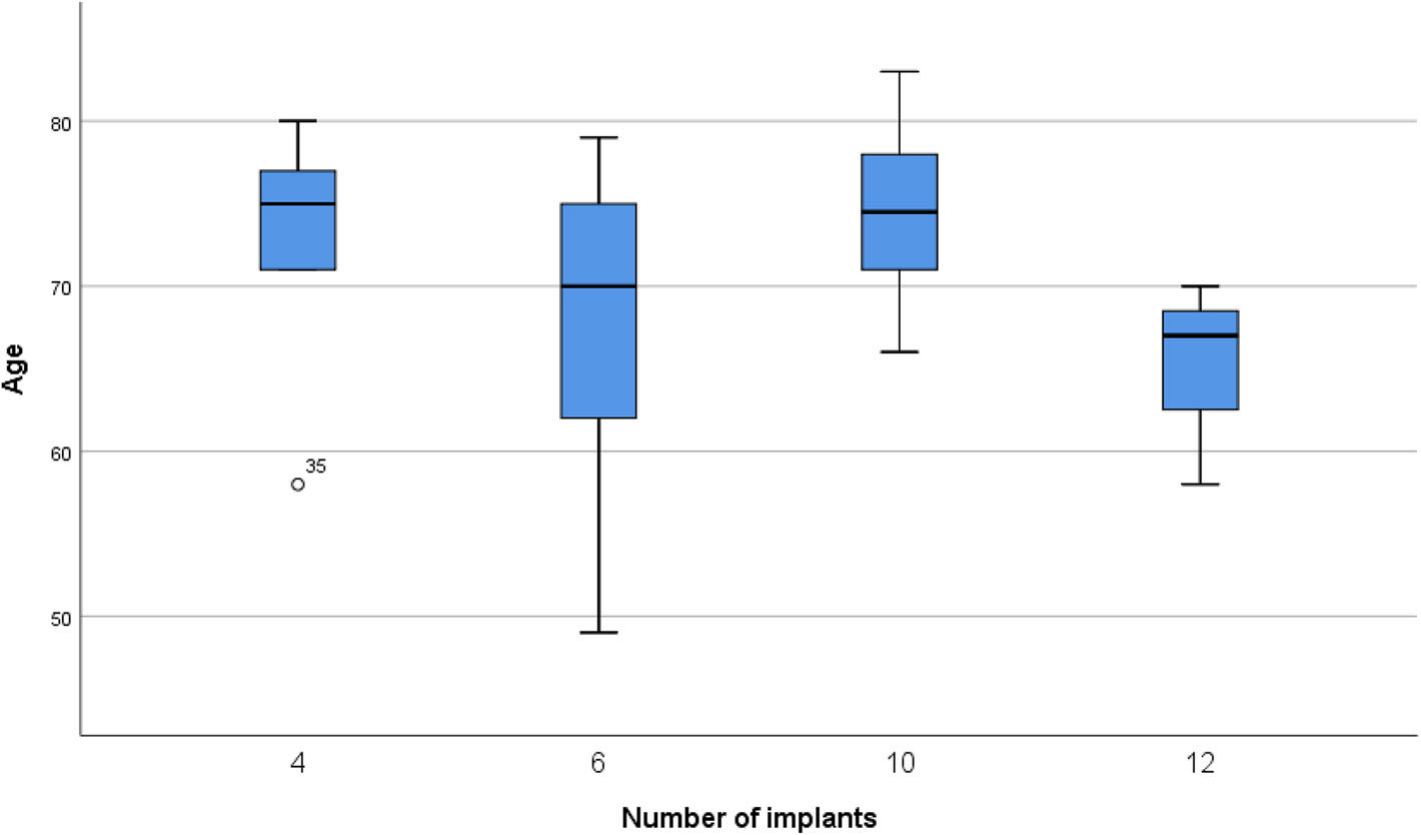
Distribution of age by number of implants

**Fig. 3:**
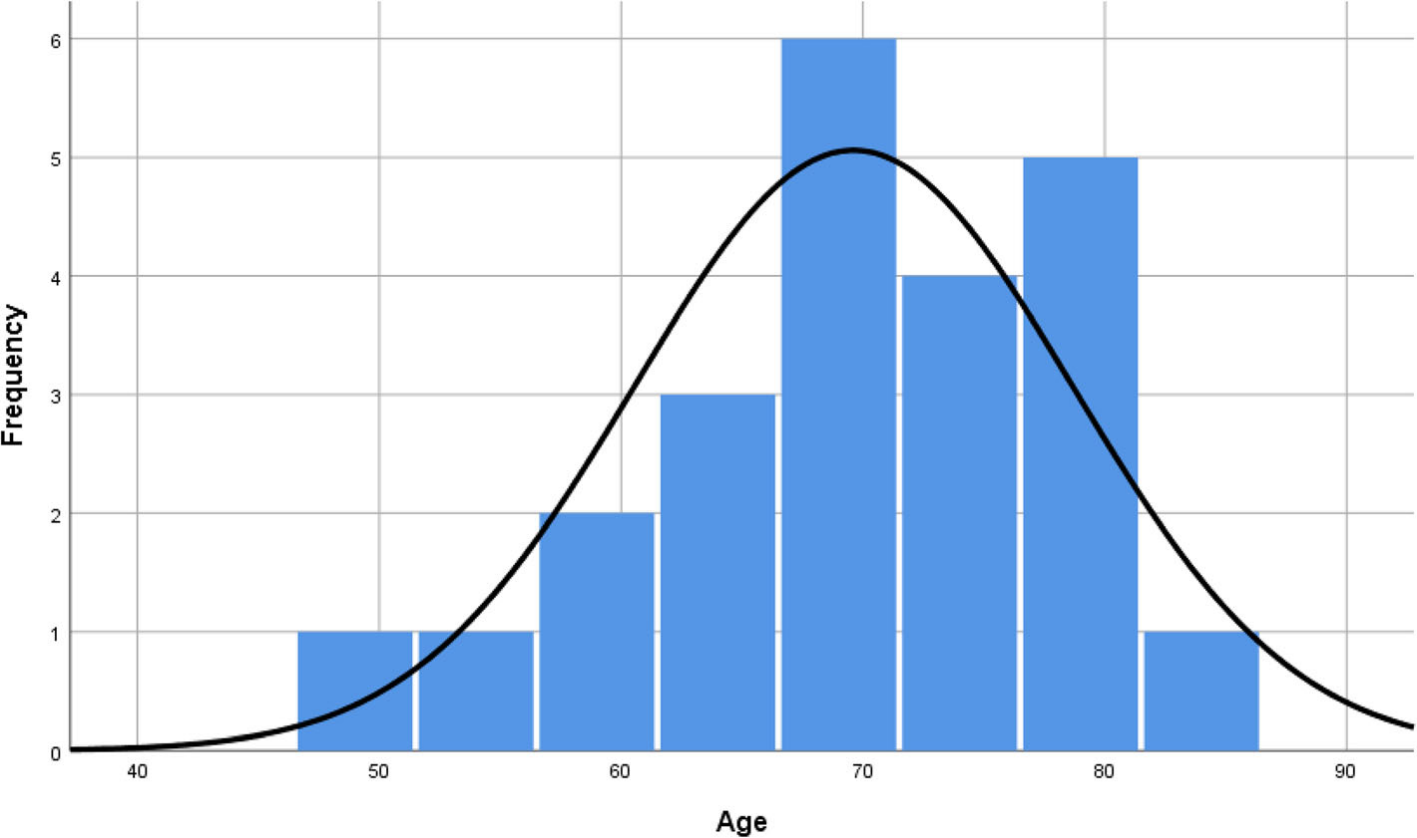
Distribution of frequency by age with exemplary normal distribution

**Fig. 4:**
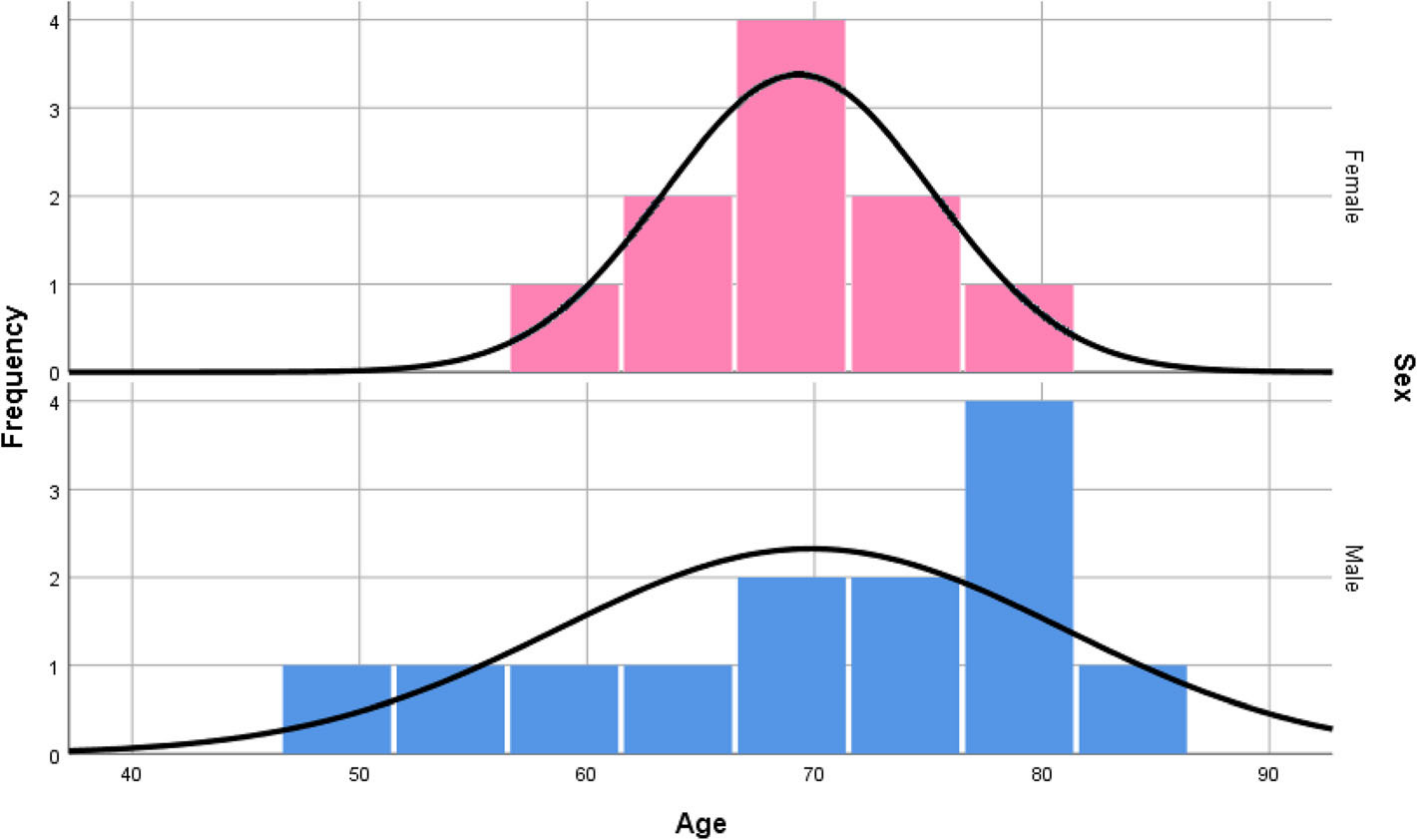
Distribution of frequency by age in female and male patients with exemplary normal distribution

A total of 170 dental implants were placed in 23 patients (Fig. 1 distribution of frequency by number of implants, divided in female and male. Most patients received 6 implants (9 patients)). All patients received an immediate resin bridge and after a minimum healing period of 3 months, the permanent restoration was performed. No implant failure or loss occurred during the follow-up period of 6 to 9 years. Patients’ age at the time of the clinical evaluation after treatment varied between 49 to 83 years at a mean of 71 years. At the time of the surgery, the patients’ age varied between 42 to 74 years at a mean of 64 years (Fig. 2 distribution of age by number of implants; Fig. 3 distribution of frequency by age with exemplary normal distribution; Fig. 4 distribution of frequency by age in female and male patients with exemplary normal distribution).

### Risk factors

Preoperatively, two patients suffered from osteoporosis and three from diabetes mellitus. No difference in crestal bone level or bleeding on probing (BOP) was found in these patients compared to the other patients of the study. Both patients admitting to nicotine abuse had positive values for BOP, which shows on-going gingival inflammation among the smokers group. Six of the 21 patients in the non-smokers group showed positive values for BOP.

### Crestal bone level

No significant changes in marginal bone levels after the follow-up period of 6 to 9 years in any patient were recorded. No crestal bone loss was detected at any of the implant sites.

### Recall compliance

All patients underwent professional cleaning appointments at a minimum of twice a year. They received rigorous training in oral hygiene. There was no significant difference of BOP between two and three times a year recall frequency.

### Periodontal status

**Fig. 5:**
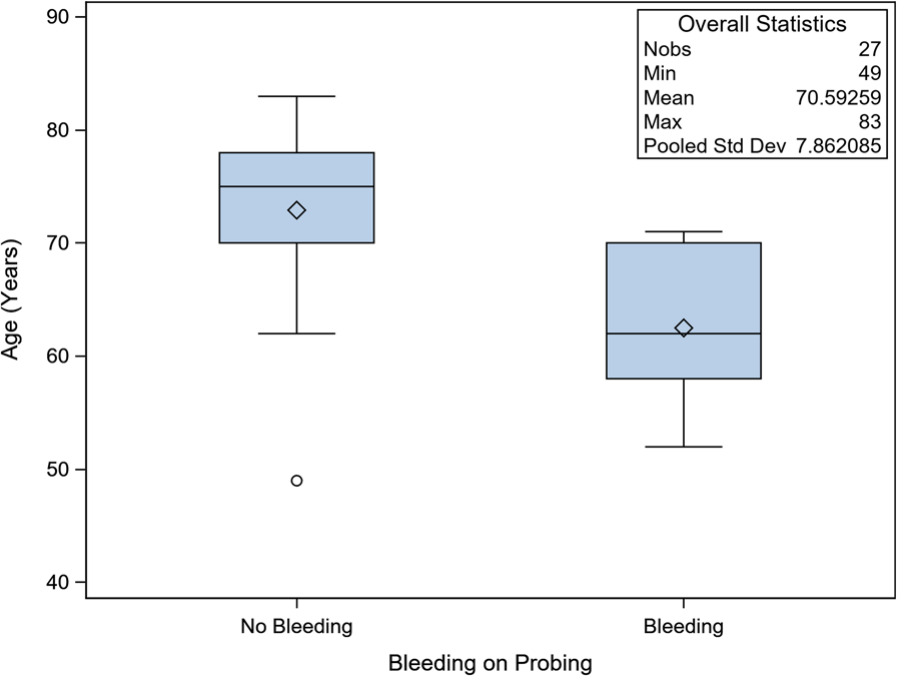
Distribution of age by bleeding-on-probing. The BOP-positive group was significantly younger

**Fig. 6:**
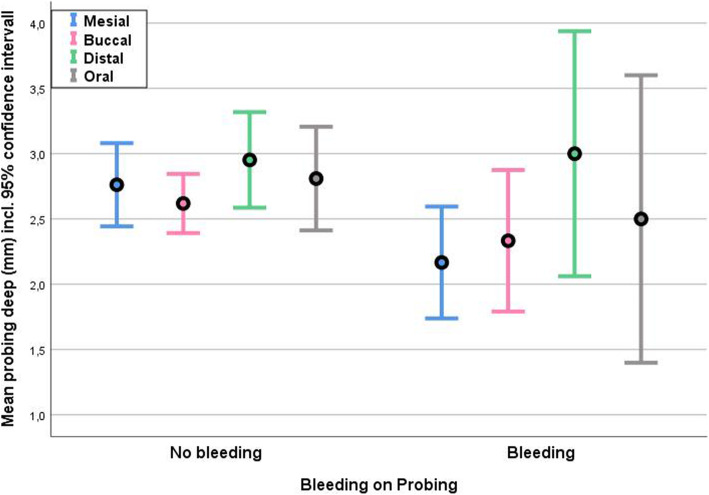
correlation between mean probing depth and bleeding on probing. In the group without bleeding on probing, the deviation of measured probing depths was significantly less

Preoperatively, 22 of 23 patients showed a medium to severe general periodontitis. A periodontal screening and recording (PSR) was done preoperatively. All panoramic x-rays showed severe horizontal bone loss before treatment. After treatment, 16 patients with preoperative positive BOP values showed negative values after the follow-up period. Six patients showed isolated positive BOP positions. One patient was already edentulous preoperatively. Patients, with more positive findings for BOP were significantly younger than patients without BOP (Fig. 5 distribution of age by bleeding-on-probing. The BOP-positive group was significantly younger). Probing depths were measured between 2 and 3 mm. The deviation of different probing depths was significantly smaller in the group without BOP (Fig. 6 correlation between mean probing depth and bleeding on probing. In the group without bleeding on probing, the deviation of measured probing depths was significantly less). Sixteen of 23 patients showed keratinized gingiva around the abutments. There was no correlation between keratinization and BOP, probing depths, or bone level. Also, the angle of implantation did not affect the BOP values.

### Prosthodontic complications

During the follow-up period, three out of 23 patients showed fracturing of a resin tooth. The fractured teeth of all three patients were repaired within 1–2 h by the dental technician.

## Discussion

No bone loss or implant loss occurred during the long-term follow-up of patients restored with the “fast&fixed”-protocol. Besides three small prosthodontic chipping cracks, which could be repaired immediately, only BOP at the implant site was detected in some of the patients (26%). Younger patients showed significantly higher positive values for BOP. The higher BOP values show a more advanced periodontitis in this group of patients and are due, possibly, to a lack of oral hygiene. Among the younger patients, a severe grade of periodontal disease may result in early tooth loss. Among the older patient group, many different factors may contribute to tooth loss including a lack of oral hygiene during youth and adolescence as well as extended, now out-dated, prosthetic restorations. Nowadays, sufficient health education resulting in better oral hygiene can prevent caries and periodontitis. Higher BOP values correlate with higher prevalence of peri-implantitis [5]. Especially among the younger patients, the clinician has to consider these in a strict maintenance care to avoid peri-implant bone loss. There is a controversy on general use of implants in patients with periodontitis. Younger patients with tooth loss are especially diagnosed with general aggressive periodontitis (GAP). Many studies report a higher risk for peri-implant bone loss in these patients; however, the long-term survival rate does not significantly differ [6]. The reported probing depths around implants in the current literature vary greatly [7]. No reliable value exists, comparing the limit of 3.5 mm in the case of tooth pockets in periodontitis [8]. In this study, probing depths of 2.5–3 mm showed significantly less BOP. Therefore, the peri-implant conditions seem most stable at these probing depths. In a prospective study with general chronic periodontitis (GCP) subjects, implants with rough surfaces showed a survival rate of 96.0% after an observation period of 11.6 years [9]. Other studies also show that implants offer a predictable long-term solution in healthy and periodontitis patients, as long as they are under strict periodontal control [10]. Several studies report similar or poorer results of implant survival rates [11–13]. However, the survival rate possibly overestimates the true state of an implant. Therefore, data like BOP, probing depths, and radiographic controlling should be recorded at the same time to provide a more detailed insight into the state of the implant. To evaluate implant success, the examinations have to include BOP, probing depths, and x-rays as mean examinations [14]. The usual implant rating after Albrektsson et al. [15] assesses single implants. In this study, implants are splinted with a metal bar or a fixed prosthesis, so the use of this rating is limited. Also, in this study, an implant was defined as successful, if it was stable and supporting a functional prosthesis [16, 17] without radiographic bone loss and absence of suppuration. Many studies investigated only the upper or the lower mandible [18, 19], and there are only a few studies, in which the patients received definitive prostheses some months later with possibly better cleaning conditions than with the first provisional bridge [20].

Neither the angulation of the implant nor the keratinization of the gingiva appeared to have an influence on BOP. By angling the implant, the implant-to-bone contact surface is increased [21], and early marginal bone loss depending on the position of the implant has been reported [22]. However, the angulation of the implants had no influence on radiographic bone loss. These results are in accordance with the results of a study by Del Fabbro and Ceresoli [12], who also reported no influence of the implant angulation on the marginal bone level. The evidence in contemporary literature linking keratinization to peri-implant health is limited [5]. In this study, no significant influence of keratinization of the mucosa on BOP or on peri-implant bone loss was found. A possible reason for this may be the good visiting habits of the patients, who come to the dental practice two or three times a year for professional cleaning and inspection. In this study, nicotine abuse showed no significant differences in comparison to the non-smoking group in all clinical parameters. However, this could be due to the very limited number of smokers in this study, especially since Sakkas et al. showed that nicotine abuse has a strong impact on implant loss [23]. Some studies report that there is an association between medication like proton pump inhibitors (PPIs) or serotonin reuptake inhibitors (SSRIs) and increased implant failure [24, 25]. PPIs inhibit the acid output to the stomach, which in turn disrupts the calcium uptake through the intestines [26, 27]. SSRIs are used to treat anxiety disorders or depression but influence the osteoblast and osteoclast balance since serotonin can regulate osteoclast activation and differentiation from hematopoietic cell precursors [28]. This negative effect on bone mineral density and bone microarchitecture results in anti-anabolic skeletal anatomy [29]. In this study, no patient received PPIs or SSRIs.

The main limitation of this study is the retrospective design. Results rely on existing radiographics, current panoramic x-rays, and periodontal measurements. Technical failures like resin fractures were recorded but not analyzed because of very low occurrence. There is evidence of increased mechanical complications in double-arch restored patients because of occlusal overload [30]. However, the authors conclude that this complication does not affect the long-term survival rates in this kind of treatment. Nevertheless, the patients of this dental practice were initially asked to avoid occlusal overload and follow a soft diet in the first 3 months, and to clean their new prosthetic restoration thoroughly twice a day with additional support by their dentist and their dental hygienist. Patients with insufficient compliance should not receive this kind of treatment. Within these limits, the treatment represents an alternative to two or multiple step procedures for immediate loading and long-term stabilities.

## Conclusion

This clinical study proves long-term stability of immediate prosthodontic rehabilitation of edentulous jaws with angulated implant-supported fixed overdentures despite compromised patients. The reduced number of implants combined with angulated positioning avoids preprosthetic augmentations and reduces treatment failure and costs. In particular, the significantly increased BOP rates among younger patients due to a possibly higher activity of remaining or recurring individual periodontitis-associated bacterial spectrum have to be further investigated. The current results suggest that the effect of nicotine abuse remains controversial. The influence of various medications in increasing older and multi-morbid patients as possible confounders in implantology should be further investigated.

## Data Availability

The datasets used and/or analyzed during the current study are available from the corresponding author on reasonable request.

## Abbreviations

BOP: Bleeding on probing
PTC: Professional tooth cleaning
PSR: Periodontal screening and recording
GAP: General aggressive periodontitis
GCP: General chronic periodontitis
PPI: Proton pump inhibitor
SSRI: Serotonin reuptake inhibitor
